# High throughput MLVA-16 typing for *Brucella *based on the microfluidics technology

**DOI:** 10.1186/1471-2180-11-60

**Published:** 2011-03-24

**Authors:** Riccardo De Santis, Andrea Ciammaruconi, Giovanni Faggioni, Silvia Fillo, Bernardina Gentile, Elisabetta Di Giannatale, Massimo Ancora, Florigio Lista

**Affiliations:** 1 Histology and Molecular Biology Section, Army Medical and Veterinary Research Center, Via Santo Stefano Rotondo 4, 00184 Rome, Italy; 2 Istituto Zooprofilattico Sperimentale dell'Abruzzo e del Molise "G. Caporale", (Istituto G. Caporale), Teramo, Italy

## Abstract

**Background:**

Brucellosis, a zoonosis caused by the genus *Brucella*, has been eradicated in Northern Europe, Australia, the USA and Canada, but remains endemic in most areas of the world. The strain and biovar typing of *Brucella *field samples isolated in outbreaks is useful for tracing back source of infection and may be crucial for discriminating naturally occurring outbreaks versus bioterrorist events, being *Brucella *a potential biological warfare agent. In the last years MLVA-16 has been described for *Brucella *spp. genotyping. The MLVA band profiles may be resolved by different techniques i.e. the manual agarose gels, the capillary electrophoresis sequencing systems or the microfluidic Lab-on-Chip electrophoresis. In this paper we described a high throughput system of MLVA-16 typing for *Brucella *spp. by using of the microfluidics technology.

**Results:**

The Caliper LabChip 90 equipment was evaluated for MLVA-16 typing of sixty-three *Brucella *samples. Furthermore, in order to validate the system, DNA samples previously resolved by sequencing system and Agilent technology, were *de novo *genotyped. The comparison of the MLVA typing data obtained by the Caliper equipment and those previously obtained by the other analysis methods showed a good correlation. However the outputs were not accurate as the Caliper DNA fragment sizes showed discrepancies compared with real data and a conversion table from observed to expected data was created.

**Conclusion:**

In this paper we described the MLVA-16 using a rapid, sophisticated microfluidics technology for detection of amplification product sizes. The comparison of the MLVA typing data produced by Caliper LabChip 90 system with the data obtained by different techniques showed a general concordance of the results. Furthermore this platform represents a significant improvement in terms of handling, data acquiring, computational efficiency and rapidity, allowing to perform the strain genotyping in a time equal to one sixth respect to other microfluidics systems as e.g. the Agilent 2100 bioanalyzer.

Finally, this platform can be considered a valid alternative to standard genotyping techniques, particularly useful dealing with a large number of samples in short time. These data confirmed that this technology represents a significative advancement in high-throughput accurate *Brucella *genotyping.

## Background

The members of the genus *Brucella *are Gram-negative, facultative intracellular bacteria responsible of a considerable human morbidity and in animals of enormous economic losses [1] due to abortion and infertility in livestock (cattle, goats, and sheep). As brucellosis is a zoonotic disease, practically all human *Brucella *infections develop from direct or indirect contact to animals. In particular, brucellosis in humans occurs as a sub-acute or chronic illness, that is generally not lethal in previously healthy patients, and can result in a wide variety of manifestations and significant morbidity if the diagnosis is unobserved and treatment is not rapidly initiated [2]. There are nine recognized species of *Brucella *[3] that differ in their host preference [4]. In particular, the nine recognized host-specific *Brucella *spp. are: *B. abortus *which preferentially infects cattle; *B. melitensis *infects sheep and goats; *B. suis *infects pigs; *B. canis *the dog; *B. ovis*, sheep and goats; *B. neotomae *the desert wood rat; *B. microti *the common vole [5]; *B.ceti*, cetaceans [6]; *B. pinnipedialis*, seals [6,7]. Recently, an additional novel species, *B. inopinata *sp., isolated from a human breast implant infection, was described [8]. Currently, the division in species and between biovars of a given species is performed using differential tests based on phenotypic characterization of lipopolysaccharide (LPS) antigens, phage typing, dye sensitivity, requirement for CO_2_, H_2_S production, and metabolic properties [9]. The genotyping of *Brucella *field strain isolated in outbreaks is an essential tool to better understand the epidemiology of the disease and to give support to the trace-back of infection sources. It is also essential to identify the presence of *Brucella *strains that can affect livestock populations and new strains that were previously considered to be exotic [10], thus improving the outcomes of the national brucellosis eradication programme. Although brucellosis has been eradicated in Northern Europe, Australia, the USA and Canada, this disease remains endemic in most areas of the world [11]. Therefore, the knowledge of the prevailing genotypes of *Brucella *spp. present in a country is an important epidemiological tool to assess the necessary steps required for the formulation of policies and strategies for the control of brucellosis in animal populations. In addition, *Brucella *spp. represent potential biological warfare agents due to the high contagious rates for humans and animals, the non-specific symptoms associated with the infection, and the fact that the organism can be readily aerosolized [12-14]. Therefore, the discrimination between natural outbreaks and/or intentional release of micro-organism agents may be of crucial importance in the context of the bioterrorism. *Brucella *species are characterised by >80% interspecies homology by DNA-DNA hybridization studies [15,16] and >98% sequence similarity by comparative genomics [17]. In fact, the sequencing of 16 S rRNA showed a 100% of identity between all of the *Brucella *spp. [18]. The simple identification of genus and, in some cases, species by PCR assays [19,20], is adequate for purposes as diagnosis of human/animal disease or identification of food contamination but not for the tracing of outbreaks or bioterrorist attack. Therefore, the development of strain typing methods is essential in order to investigate the source of an epidemic event. Molecular DNA technology such as repetitive intergenic palindromic sequence-PCR (REP-PCR) [21], random amplified polymorphic DNA-PCR (RAPD-PCR) [22], arbitrary primed-PCR (AP-PCR) [23], amplified fragment length polymorphism (AFLP) [24], single nucleotide polymorphism (SNP) [25,26], and polymerase chain reaction-restriction fragment length polymorphism (PCR-RFLP) [27] has been employed to sub-type *Brucella *spp.

In the last years the variable number of tandem repeats (VNTR), allelic hypervariability related to variation in the number of tandemly repeated sequences, were used for the discrimination of bacterial species that display very little genomic diversity. Polymorphic tandem repeat loci have been identified by analysing published genome sequences of *B. melitensis *16 M, *B. suis *1330, and *B. abortus *9-941 [16,28]. Schemes based on multiple locus VNTR analysis (MLVA) were tested. In *Brucella*, MLVA schemes with 21 loci (MLVA-21), 15 and 16 loci (MLVA-15 and MLVA-16) were published [12,16,29]. The authors used a subset of loci that preserved the clusters corresponding to classical species, comprising markers with repeat unit sizes of 9 bp or greater and good species identification capability ('minisatellites') and markers with repeat unit sizes of up to 8 bp and higher discriminatory power ('microsatellites') [30]. The MLVA band profiles may be resolved by different techniques ranging from low cost manual agarose gels to the more expensive capillary electrophoresis sequencing systems. The most frequently used method is the agarose gel. Recently, a more rapid and inexpensive method based on the Lab on a chip technology has been proposed [31]. This miniaturized platform for electrophoresis applications is able to size and quantify PCR fragments, and was previously used for studying the genetic variability of *Brucella *spp. [32]. Recently a new high throughput micro-fluidics system, the LabChip 90 equipment (Caliper Life Sciences), was developed. This platform can be considered particularly useful when dealing with a large number of samples in short time. Therefore we evaluated the LabChip 90 system for MLVA typing of *Brucella *strains applying the selected subset of 16 loci proposed by Al-Dahouk et al. [12] to fifty-three field isolates and ten DNA samples provided in 2006 for *Brucella suis *ring-trial. Furthermore, twelve DNA samples, provided in 2007 for a MLVA VNTR ring trial and seventeen human *Brucella *isolates whose MLVA fingerprinting profiles were previously resolved [32,33], were *de novo *genotyped.

## Results

By means of MLVA-16 on LabChip 90 (Caliper Life Sciences) sixty-three DNA samples, fifty-three field isolates of *Brucella *(Table 1) and ten DNA provided for *Brucella suis *ring-trial, were analysed for investigating a broader number of loci. In order to set up the system, DNA samples, previously genotyped by sequencing system and Agilent technology [32,33], were reanalyzed. DNA from all ninety-two isolates was amplified at 16 loci (MLVA-16 typing assay) to generate multiple band profiles. The LabChip 90 equipment acquires the sample in less than a minute and the analysis of 96 samples in less than an hour. After PCR amplification 5 μl of each reaction was loaded into a 96-well plate and the amplification product size estimates were obtained by the LabChip Gx Software. The data produced by the Caliper system showed band sizing discrepancies compared with data obtained from other electrophoresis platforms. Therefore a conversion table that would allow the allocation of the correct alleles to the range of fragment sizes was created. The table contained for each locus the expected size, the range of observed sizes, including arithmetical average ± standard deviation, and the corresponding allele (Table 2). The variability range for each allele was established experimentally by the analysis of different strain amplification products. Furthermore, in order to look at intra- and interchip variability, each allele was analyzed by repeating five times the analysis on the same chip and different chips. The comparison of the average and standard deviations obtained by the analysis of the intra- and interchip variability by t-test (confidence of interval 95%) shown a P value > 0.005 (data not shown).

**Table 1:** The fifty-three strains provided by Istituto Zooprofilattico Sperimentale dell'Abruzzo e del Molise - G. Caporale-(Istituto G. Caporale).

Samples	Species-biovar according MLVA Database Genotypinga	Year	Host	Geographic origin
BruIT200	B.melitensis biovar 3	2002	human	Sardinia, Italy
BruIT201	B.abortus biovar 1	2002	bovine	Piemonte, Italy
BruIT202	B.melitensis biovar 3	2002	bovine	Lazio, Italy
BruIT203	B.abortus biovar 1	2002	bovine	Lazio, Italy
BruIT204	B.abortus biovar 3	2002	bovine	Piemonte, Italy
BruIT205	B.melitensis biovar 3	2002	water buffalo	Campania, Italy
BruIT206	B.melitensis biovar 3	2002	water buffalo	Campania, Italy
BruIT207	B.abortus biovar 1	2003	water buffalo	Campania, Italy
BruIT208	B.melitensis biovar 3	2003	milk	Emilia-Romagna, Italy
BruIT209	B.melitensis biovar 3	2003	bovine	Abruzzo, Italy
BruIT210	B.abortus biovar 3	2001	bovine	Piemonte, Italy
BruIT211	B.abortus biovar 3	2001	bovine	Piemonte, Italy
BruIT212	B.abortus biovar 3	2002	bovine	Piemonte, Italy
BruIT213	B.abortus biovar 3	2007	bovine	Italy
BruIT214	B.abortus biovar 3	2002	bovine	Piemonte, Italy
BruIT215	B.melitensis biovar 3	2001	ovine	Lazio, Italy
BruIT216	B.melitensis biovar 3	2001	ovine	Lazio, Italy
BruIT217	B.melitensis biovar 3	2001	water buffalo	Lazio, Italy
BruIT218	B.melitensis biovar 3	2002	bovine	Campania, Italy
BruIT219	B.melitensis biovar 3	2001	wild boar	Campania, Italy
BruIT220	B.melitensis biovar 3	2001	bovine	Piemonte, Italy
BruIT221	B.melitensis biovar 3	2001	ovine	Piemonte, Italy
BruIT222	B.melitensis biovar 3	2001	ovine	Lazio, Italy
BruIT223	B.melitensis biovar 3	2001	ovine	Lazio, Italy
BruIT224	B.abortus biovar 3	2001	bovine	Lazio, Italy
BruIT225	B.abortus biovar 3	2001	bovine	Piemonte, Italy
BruIT226	B.melitensis biovar 3	2001	human	Lazio, Italy
BruIT227	B.suis biovar 2	2003	hare	Emilia-Romagna, Italy
BruIT228	B.suis biovar 2	2003	hare	Emilia-Romagna, Italy
BruIT239	B.abortus biovar 3	2008	bovine	Molise, Italy
BruIT240	B.abortus biovar 3	2008	bovine	Molise, Italy
BruIT241	B.abortus biovar 3	2008	bovine	Molise, Italy
BruIT242	B.abortus biovar 3	2008	bovine	Molise, Italy
BruIT243	B.abortus biovar 3	2008	bovine	Molise, Italy
BruIT244	B.abortus biovar 3	2008	bovine	Molise, Italy
BruIT245	B.abortus biovar 3	2007	water buffalo	Campania, Italy
BruIT246	B.melitensis biovar 3	2007	water buffalo	Campania, Italy
BruIT247	B.abortus biovar 3	2007	bovine	Calabria, Italy
BruIT248	B.abortus biovar 3	2007	water buffalo	Puglia, Italy
BruIT249	B.abortus biovar 3	2009	bovine	Campania, Italy
BruIT250	B.abortus biovar 3	2009	bovine	Calabria, Italy
BruIT251	B.abortus biovar 3	2009	bovine	Calabria, Italy
BruIT252	B.abortus biovar 6	2009	bovine	Calabria, Italy
BruIT253	B.abortus biovar 6	2009	ovine	Puglia, Italy
BruIT254	B.melitensis biovar 3	2001	bovine	Piemonte, Italy
BruIT255	B.abortus biovar 3	2002	bovine	Piemonte, Italy
BruIT256	B.suis biovar 2	2002	bovine	Piemonte, Italy
BruIT257	B.suis biovar 2	2001	ovine	Lazio, Italy
BruIT258	B.suis biovar 2	2005	water buffalo	Campania, Italy
BruIT259	B.suis biovar 2	2002	wild boar	Piemonte, Italy
BruIT260	B.abortus biovar 1	2007	bovine	Campania, Italy
BruIT261	B.abortus biovar 3	2007	bovine	Italy
BruIT262	B.abortus biovar 1	2007	bovine	Calabria, Italy

^a^MLVA bank for bacterial genotyping http://mlva.u-psud.fr/[35].

**Table 2:** Comparison between *Brucella *product sizes estimated by LabChip GX software (Observed size) and actual sizes obtained by direct sequencing of the PCR product or data available in Genbank (Expected size).

PCR	Locus (UL bps)^a^	Allele	Expected size	Observed size	x ± s^b^
Singleplex 1	Bruce08 (18)	2	312		
		3	330	346-359	352,63 ± 5,37
		4	348	369-383	376 ± 4,62
		5	366	385-410	399,09 ± 6,58
		6	384	411-434	419,29 ± 6,71

Singleplex 2	Bruce43 (12)	1	170	179-188	183,17 ± 2
		2	182	191-200	196,07 ± 2,32
		3	194		

Singleplex 3	Bruce12 (15)	7	302		
		8	317		
		9	332		
		10	347	359-369	362,8 ± 3,7
		11	362	379-388	384,13 ± 3,64
		12	377	390-400	395,16 ± 3,05
		13	'392	409-420	413 ± 2,55
		14	407	424-433	428,82 ± 3,05
		15	422	434-440	438,25 ± 2,87
		17	452		

Singleplex 4	Bruce18 (8)	3	130	143	
		4	138	150-157	153,57 ± 2,64
		5	146	159-162	160,33 ± 1,37
		6	154	164-176	171,62 ± 2,95
		7	162	178-184	181,65 ± 1,53
		8	170	187-194	191 ± 2,24
		9	178		

Singleplex 5	Bruce11 (63)	2	257	266-270	268 ± 2,82
		3	320	321-344	337,82 ± 4,31
		4	383	407-422	410,52 ± 3,56
		6	509	504-536	515,8 ± 12,52
		8	635	623-649	639,6 ± 8,71
		9	698	680-724	696,67 ± 15,6
		12	887		
		15	1076		

Singleplex 6	Bruce21 (8)	5	140		
		6	148	162	
		7	156	178-179	178,5 ± 0,71
		8	164	180-186	182,55 ± 1,19
		9	172	192-199	194,05 ± 1,94

Singleplex 7	Bruce06 (134)	1	140	151	
		2	274	282-294	285,9 ± 3,33
		3	408	429-454	439,89 ± 6,04
		4	542	518-624	575,4 ± 24,92

Singleplex 8	Bruce42 (125)	1	164	172-198	175,1 ± 3,13
		2	289	279-298	288,88 ± 2,14
		3	414	420-442	428,27 ± 6,18
		4	539	504-569	529,31 ± 14,1
		5	664	642-647	644 ± 2,64
		6	789	695-763	726,4 ± 22,02
		7	914		

Singleplex 9	Bruce45 (18)	2	133		
		3	151	156-169	162.01 ± 1,93
		4	169		
		5	187	196-206	198,95 ± 2,63

Singleplex 10	Bruce55 (40)	1	193	204-209	207,05 ± 1,67
		2	233	243-259	248,36 ± 4,09
		3	273	275-308	282,85 ± 2,5
		4	313	327	
		5	353		
		6	393	418-422	420,25 ± 1,7
		7	433		

Singleplex 11	Bruce30 (8)	2	119	130	
		3	127	132-144	139,29 ± 2,11
		4	135	146-152	148,87 ± 1,7
		5	143	155-160	157,77 ± 1,78
		6	151	165-169	167 ± 2
		7	159	174	
		8	167		
		9	175		
		10	183	205-206	202,25 ± 0,5
		11	191		
		12	199		

Singleplex 12	Bruce04 (8)	2	152	161-164	162.5 ± 2.1
		3	160	169-175	171.6 ± 2
		4	168	177-182	179.1 ± 1.3
		5	176	185-191	187.3 ± 1.8
		6	184	194-198	195.7 ± 1.3
		7	192	201-207	203.4 ± 2.2
		8	200	213-214	213.7 ± 0.6
		9	208	219-222	220.5 ± 2.1
		10	216	241	
		11	224	248-254	250.2 ± 2.4
		12	232		
		13	240		
		14	248		
		15	256		
		17	272		
		18	280		
		19	288		
		20	296		
		22	312		

Singleplex 13	Bruce07 (8)	2	134		
		3	142		
		4	150	150-154	151.9 ± 1.5
		5	158	157-162	159.8 ± 1.4
		6	166	166-171	168.1 ± 1.4
		7	174	175-178	176.8 ± 1
		8	182	183-186	184.4 ± 1.1
		9	190	192-195	195 ± 1.5
		10	198	200	
		11	206		
		12	214		
		13	222		
		14	230		

Singleplex 14	Bruce 09 (8)	3	124	131-140	135,52 ± 2,6
		4	132	147	
		5	140	155-158	156,33 ± 1,52
		6	148	162-167	165,4 ± 1,89
		7	156	172-177	174,42 ± 1,19
		8	164	182-187	184,42 ± 1,61
		9	172	191-198	193,75 ± 2,5
		10	180	201-203	202,12 ± 0,83
		11	188	209-212	210,75 ± 1,25
		12	196	220	
		13	204	228-230	228,66 ± 1,15
		14	212		
		15	220		
		16	228	249-255	252,66 ± 3,21
		17	236		
		18	244	266-271	268,85 ± 1,86
		19	252		
		20	260		
		22	276		
		23	284		
		24	292		

Singleplex 15	Bruce 16 (8)	2	144	153-157	154,9 ± 1,59
		3	152	158-166	163,04 ± 2,38
		4	160	167-172	168,53 ± 1,66
		5	168	177-185	181,52 ± 2
		6	176	186-194	189,83 ± 2,55
		7	184	199-203	200,8 ± 1,4
		8	192	207-209	207,66 ± 1,15
		9	200	216-219	217,37 ± 1,18
		10	208	224-227	224,75 ± 1,5
		11	216	231	
		12	224	242-248	244,75 ± 2,5
		14	240		
		15	248		

Singleplex 16	Bruce 19 (6)	4	79		
		5	85		
		6	91		
		15	145		
		16	151		
		18	163	173-177	175 ± 1,4
		19	169	180-183	182,5 ± 0,5
		20	175	184-188	186 ± 1,8
		21	181	189-193	190,6 ± 1,2
		22	187	194-201	197,9 ± 1,1
		23	193	202	
		25	205		

^a ^Unit Length size

^b ^Arithmetic average (x) ± standard deviation (σ) of the observed sizes

The required precision is directly related to the repeat unit size of the loci. Only data with a standard deviation lower than the 50% of the repeat unit size were considered valid. The LabChip 90 equipment MLVA-16 products were separated and DNA fragment sizes were correlated to the alleles by the conversion table. Generally, close alleles were not observed to overlap allowing to assign the correct allele to each observed value. However, the markers Bruce 08, Bruce 21, Bruce 16 and Bruce 19 showed continuity between some neighboring range which may lead to incorrect assignment of allele to the observed value (Table 2). The identified species were compared with the results of the previous analysis [32,33], obtaining a full concordance for 15 markers while the marker Bruce 19 did not show agreement with the results obtained by the different analysis systems. For the loci including alleles spanning into ambiguous ranges, we performed sequencing of the amplicons showing on Caliper maximum or minimum allele values. Furthermore we performed some random sequencing of the amplicons obtaining a confirmation of the correct assignment (data not shown).

## Discussion

Many methods have been developed to differentiate *Brucella *strains but MLVA currently represents one of the most promising technologies regarding the epidemiology of bacteria with a high genetic homogeneity, such as *Brucella *ssp. In 2003 Bricker et al [28] published a MLVA based on eight locus scheme. In 2006 Whatmore et al [16] described a new scheme that included the eight of the original loci of Bricker as well as an additional 13 newly VNTR loci to give a 21 locus scheme, VNTR-21, that allowed to provide some resolution at the species level. In the same year a scheme labelled MLVA-15, based on a subset of 15 loci that comprises 8 markers with good species identification capability and 7 with higher discriminatory power, was published [29], and followed by MLVA-16, a slight modification of MLVA-15 [12]. The different alleles, amplified by standard PCR techniques, can be analysed by several electrophoretic techniques as agarose gel, or capillary electrophoresis sequencing. In this paper the attention was addressed on the LabChip 90 equipment (Caliper), a platform based on microfluidics technology specifically developed for measuring the length of DNA fragments and that do not require fluorescent primers. This electrophoresis machine represents a compromise between the more expensive capillary electrophoresis apparatus and the traditional agarose gel electrophoresis. In spite of a lower precision respect to the automated capillary electrophoresis, the ability to acquire 96 amplification product sizes in less than a hour represent an increased time-reduction over the traditional ethidium bromide slab gel electrophoresis, with 40-50 amplification product sizes for the same analysed markers acquired in a higher time [34]. The LabChip 90 represents also a significant improvement respect to other microfluidics systems as e.g. the Agilent 2100 bioanalyzer (Agilent Technologies, Palo Alto, Ca). In effect the LabChip 90 allows performing the strain genotyping in a time equal to one sixth respect to Agilent. Furthermore this system requires less handling as a single plate can be read directly after the PCR reaction, while the Agilent equipment needs a manual charge of the single PCR products for each single chip well. Finally, the LabChip GX software improves efficiency of data acquiring by automating the data flows. In fact, the software allows to export the summary of analysis results to a spreadsheet application, with the consequent elimination of the paper-based flows. As described previously [31,32] the sizing proposed by the Lab on chip technology does not correspond to the real size, resulting in a shift of a variable value (offset) respect to the real size estimated by sequencing. Therefore, a correspondence table which allows for each range of observed values to assign the expected size and corresponding allele (Table 2) was created. We did not observe in general the overlap among close alleles, allowing to unambiguously assign the correct allele to each observed value. However, for some contiguous alleles we observed a continuity between ranges which may lead to incorrect assignment of allele to the observed value (Table 2). Furthermore, the instrument was not in agreement with the results obtained by the different analysis systems for the marker Bruce 19. The reduced discriminatory ability could be due to the different resolution achieved by such platform related to the fragment sizes (routinely ± 10% in a 150-500 -bp range, ± 15% in a 100-150 -bp range and in a 500-1500 -bp range and ± 20% in a 1500-5000 -bp range). However, the comparison of the results obtained by the MLVA-16 method on the Caliper LabChip 90 platform and those previously resolved by capillary electrophoresis sequencing system and the Lab on a chip technology (Agilent Technologies) showed a good size correlation. Therefore, this platform can be considered a valid alternative to standard genotyping technique, particularly useful dealing with a large number of samples in short time.

## Conclusion

In this paper we evaluated high throughput system as the LabChip 90 for MLVA-16 typing of *Brucella *strains. The MLVA typing data obtained on this equipment showed accurate correlation for those obtained by capillary electrophoresis sequencing and the Agilent 2100 Bioanalyzer, with the exception of Bruce 19. This new platform represents a significant improvement of the genotyping techniques in terms of turnaround times and computational efficiency.

## Methods

### *Brucella *strains and DNA extraction

In this study fifty-three field isolates submitted for typing by the Istituti Zooprofilattici Sperimentali to the National Reference Laboratory for brucellosis at the Istituto Zooprofilattico Sperimentale dell'Abruzzo e del Molise-G. Caporale (Istituto G. Caporale) during the 2001-2008 period (Table 1), ten DNA samples, collected in UK, provided at the Istituto Zooprofilattico Sperimentale dell'Abruzzo e del Molise-G. Caporale (Istituto G. Caporale) for *Brucella suis *ring-trial 2006 (COST 845-Brucellosis in man and animals), seventeen *Brucella *strains isolated from Sicilian hospitalized patients with acute brucellosis [33], and twelve DNA samples, provided by Dr. Falk Melzer for the Ring trial *Brucella *2007 [32], were analysed. The provided DNA samples were extracted by Maxwell 16 Cell DNA purification kit (Promega), according to the manufacturer's instructions.

### VNTR amplification

VNTR amplifications were performed according to the method described by Le Flèche et al. [29] and then adapted by Al Dahouk et al [12]. Sixteen sets of primers previously proposed were used in sixteen singleplex: Bruce06, Bruce08, Bruce11, Bruce12, Bruce42, Bruce43, Bruce45, Bruce55 (panel 1), Bruce18, Bruce 19, Bruce21, Bruce04, Bruce07, Bruce09, Bruce16, and Bruce30 (panel 2). Amplification reaction mixtures were prepared in 15 μl volumes using 1U FastStart polymerase Taq (Roche) and containing 1 ng of DNA, 1 × PCR Roche reaction buffer (10 mM Tris-HCl, 2,5 mM MgCl2, 50 mM KCl pH 8.3), 0.2 mM dNTPs (Roche) and 0.3 μM of each flanking primer. The amplification was run in a Peltier Thermal Cycler DNA Engine DYAD (MJ Research) thermocycler as follows: an initial heating at 95°C for 5 min, 35 cycles denaturation at 95°C for 30 sec, annealing at 60°C for 30 sec and extension at 70°C for 60 sec. A final extension was performed at 70°C for 5 min [32].

### MLVA-16 analysis

The amplification was performed in 96-well or 384-well PCR plates. The chip was prepared according to manufacturer recommendations (Caliper HT DNA 5 K Kit). Each chip contains 5 active wells: 1 for the DNA marker and 4 for gel-dye solution. For each run it was prepared also a strip well with the ladder (containing eight MW size standards of 100 300 500 700 1100 1900 2900 4900 bp) that was inserted into the appropriate groove of the instrument. The number of samples per chip preparation is 400, equivalent or four 96-well plates or one 384-well plate. After gel preparation, the sample plate was loaded into the plate carrier attached to the robot of the Caliper LabChip 90. During the separation of the fragments, the samples were analyzed sequentially and electropherograms, virtual gel images and table data were shown. Amplification product size estimates were obtained by using the LabChip GX (Caliper Life Sciences). The software allows importing the data to a spreadsheet software and subsequently to the conversion table that, by a special macro set up by our laboratory, allows to assign each size to the corresponding allele. The maximum and minimum value of the observed sizes for each allele was thus established experimentally while the arithmetic average and the corresponding standard deviation (Table 2) were calculated by a statistical function.

### Sequencing analysis

The PCR amplicons were purified and sequenced by CEQ 8000 automatic DNA Analysis System (Beckman-Coulter, Fullerton, CA, USA) using a commercial Kit (GenomeLab™ DTCS-Quick Start Kit, Beckman-Coulter) according to the manufacturer instructions.

## Authors' contributions

RDes and ACia did the set up of the *Brucella *MLVA-16 assay. Rdes, ACia and CMa participated to typing work. FL, EDG and MAn did the error checking analysis. SFi and GFa did various sequence analysis. FL, BGe and RDes were in charge of the database and clustering analyses. FL, MAn, and RDes conceived the study. FL and RDes wrote the report. All authors read and approved the final manuscript
